# Direct Reprogramming of Cardiac Fibroblasts to Repair the Injured Heart

**DOI:** 10.3390/jcdd8070072

**Published:** 2021-06-22

**Authors:** Emma Adams, Rachel McCloy, Ashley Jordan, Kaitlin Falconer, Iain M. Dykes

**Affiliations:** 1 Pharmacy and Biomolecular Science, Liverpool John Moores University, Liverpool L3 3AF, UK; emmaadams1@live.com (E.A.); rachel.mccloy@hotmail.com (R.M.); ashleyjordan1997@hotmail.co.uk (A.J.); kdafalconer@gmail.com (K.F.); 2 Liverpool Centre for Cardiovascular Science, Liverpool John Moores University, Liverpool L3 3AF, UK

**Keywords:** reprogramming, stem cell, regenerative medicine, induced pluripotent stem cell, myocardial infarction, heart failure

## Abstract

Coronary heart disease is a leading cause of mortality and morbidity. Those that survive acute myocardial infarction are at significant risk of subsequent heart failure due to fibrotic remodelling of the infarcted myocardium. By applying knowledge from the study of embryonic cardiovascular development, modern medicine offers hope for treatment of this condition through regeneration of the myocardium by direct reprogramming of fibrotic scar tissue. Here, we will review mechanisms of cell fate specification leading to the generation of cardiovascular cell types in the embryo and use this as a framework in which to understand direct reprogramming. Driving expression of a network of transcription factors, micro RNA or small molecule epigenetic modifiers can reverse epigenetic silencing, reverting differentiated cells to a state of induced pluripotency. The pluripotent state can be bypassed by direct reprogramming in which one differentiated cell type can be transdifferentiated into another. Transdifferentiating cardiac fibroblasts to cardiomyocytes requires a network of transcription factors similar to that observed in embryonic multipotent cardiac progenitors. There is some flexibility in the composition of this network. These studies raise the possibility that the failing heart could one day be regenerated by directly reprogramming cardiac fibroblasts within post-infarct scar tissue.

## 1. Introduction

Myocardial infarction (MI; commonly known as a heart attack) is one of the leading causes of mortality and morbidity in the Western world. MI is caused by occlusion of a coronary artery leading to ischaemic myocardial cell death. Remodelling of the myocardium following a myocardial infarction results in the formation of a fibrotic scar leading to subsequent heart failure, and this is a significant cause of mortality amongst survivors of myocardial infarction [1,2,3].

Recent advances in regenerative medicine have raised the possibility that this scar tissue itself could be used as the basis for the restoration of function. We now understand that cell fate is not irreversibly determined during embryonic development but that the epigenetic mechanisms that maintain cell fate in a structure such as the heart can be reversed using the tools of molecular biology. Fifteen years ago, it was demonstrated that a fully determined somatic cell, such as a skin fibroblast, could be converted by driving expression of four transcription factors into an induced pluripotent state resembling an embryonic stem cell, from which any somatic cell type could subsequently be differentiated [4]. This technology, in turn, subsequently led to efforts to directly convert one somatic cell type into another in a process known as transdifferentiation [5,6,7]. These efforts offer the possibility that in myocardial infarction patients, fibrotic scar tissue could one day be induced to transdifferentiate into functional myocardium, reversing heart failure and improving both quality of life and survival rates.

In this review, we will describe the molecular mechanisms that specify the major cardiac cell types in the embryo and use this as a framework to understand attempts to change cell fate in regenerative medicine. Many transcription factors, as well as other regulators of gene expression such as micro RNA, have been repurposed for therapeutic applications in an attempt to convert the scar tissue that forms during remodelling following a myocardial infarction into contractile tissue to restore the function of the heart. We will provide an overview of the many methods used and comment on the current state of the field.

## 2. Embryonic Development of the Heart

### 2.1. Cellular Composition of the Adult Heart

Cardiomyocytes, the force-producing contractile muscle of the heart, make up about 70–85% of the volume of the heart but only around a third of the total cell number [8]. The adult human heart contains as many as nine major cell types and 20 sub-types [8]. Cardiomyocytes are characterised by expression of contractile filaments within the sarcomere comprising proteins such as myosin heavy chain (MYH7, MYH6), cardiac alpha actin (ACTC1), alpha actinin (ACTN1), myomesin (MYOM1, MYOM2), titin (TTN), and troponin (TNNT2, TNNI3, TNNC1). The regulation of these genes is complex, and many exhibit isoform-specific changes during cardiomyocyte maturation [9,10,11]. Cardiomyocytes within the adult heart are not a homogenous population. There are, for example, differences between ventricular and atrial cardiomyocytes, while specialised cardiomyocytes form the conduction system.

Non-cardiomyocytes make up the majority of cells within the heart. These are essential for its function, providing structure through secretion of the extracellular matrix, providing the vascular supply and mediating homeostasis. Recent advances in single-cell RNA sequencing have allowed us to map the diversity of cell types within the heart based on transcriptional profiling and to follow changes during embryonic development and in disease [12,13]. Estimates of the precise proportions of each cell type vary, and there may be species differences [14].

Cardiac fibroblasts provide structural support through secretion of type I fibrillar collagen, forming a matrix in which is embedded the cardiomyocytes [15]. Cardiac fibroblasts also have a remarkable ability to sense and respond to stress or injury (discussed below). Fibroblasts are a molecularly heterogeneous population of cells with a gene expression profile overlapping multiple cell types^16^. Indeed, a lack of specific markers has impaired their study [16]. Genes expressed by fibroblasts include THY1/CD90, the intermediate filament vimentin and various collagens. Surprisingly, cardiac fibroblasts also express many transcription factors shared with cardiogenic precursors, including GATA4, NKX2.5, and MEF2C [17].

Other non-cardiomyocyte cell types include those comprising the vascular system of the heart (endothelium, pericytes, and vascular smooth muscle) and the immune system, including all types of leukocytes, as well as a large population of resident macrophages [18].

### 2.2. Fate Specification and Lineage Restriction during Embryonic Heart Development

During embryogenesis, the developmental potential (potency) of a cell is gradually restricted as it becomes committed to a specific lineage. The embryo is derived from pluripotent stem cells residing within the inner cell mass of the blastocyst. These cells contain open chromatin in an unmethylated state. As development proceeds, alternative cell fates are permanently closed through DNA methylation [19] and chromatin remodelling [20], resulting in the production of heterochromatin. Cell fate is largely regulated by transcription factors that recruit epigenetic modifiers such as chromatin remodelling complexes.

One of the earliest events in embryonic development is the lineage restriction event producing the three primary germ layers: the endoderm, mesoderm, and ectoderm. Most cardiovascular cell types are derived from the mesoderm germ layer. Within the mesoderm lineage, the next fate restriction divides this population into paraxial, intermediate, and lateral plate mesoderm; the lateral plate mesoderm is then physically divided by formation of the coelom into splanchnic (or visceral) and somatic mesoderm [21].

**Figure 1:**
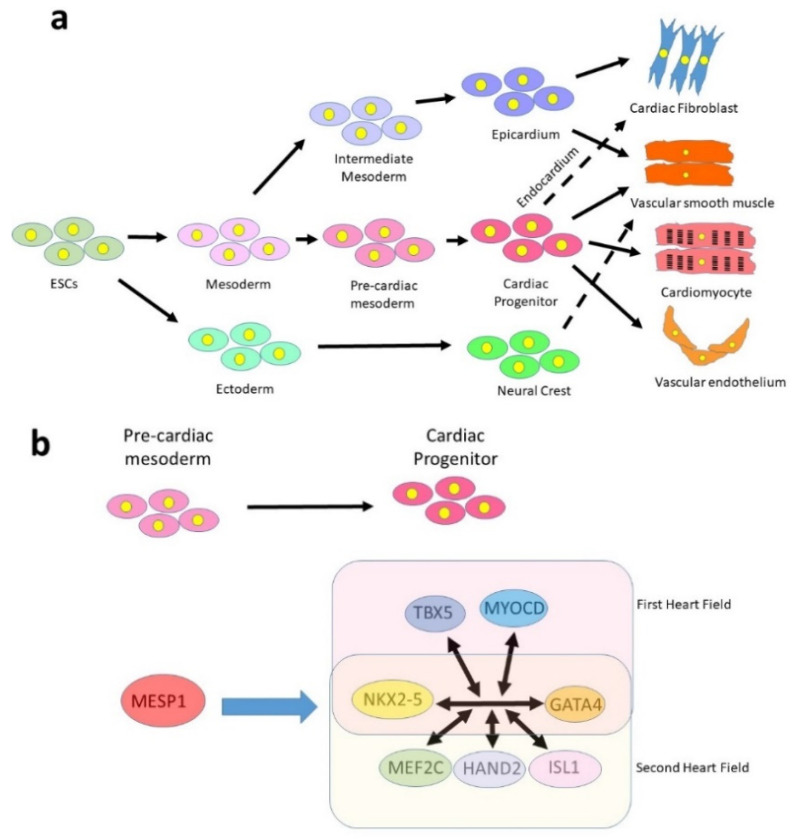
**Embryonic development of the cardiovascular lineages.** (**a**) Cardiomyocytes are derived from a multipotent cardiac progenitor of mesodermal origin, which also produces vascular smooth muscle, endothelium and cardiac fibroblasts (via the endocardium). Most cardiac fibroblasts, however, are derived from the epicardium, which can also generate smooth muscle. The neural crest, of ectodermal origin, contributes some smooth muscle as well as other cell types not shown in the diagram (valve interstitial cells, neurons and great artery fibroblasts). (**b**) Simplified version of the cardiogenic network that specifies cardiogenic progenitors. GATA4 and NKX2-5 are expressed in all progenitors, while other factors are expressed only in the first or second heart fields.

Cardiomyocytes have a single embryonic origin, being derived from a region of the splanchnic mesoderm known as the pre-cardiac mesoderm (Figure 1a). The transcription factor MESP1 is the earliest marker of multipotent cardiac progenitors and is expressed in some cells as they migrate from the primitive streak [22]. The precise function of MESP1 is unclear [23,24,25,26], but there is no master regulator of the cardiomyocyte lineage akin to MYOD in skeletal muscle and MESP1 alone is insufficient to specify cardiac progenitors [27]. Specification appears, instead, to depend not on a single transcription factor, but on a network of interacting factors activated downstream from MESP1 [28,29,30,31]. MESP1+ cells give rise at different timepoints to two distinct progenitor populations, the first and second heart fields [32], which may be distinguished on the basis of a distinct but overlapping expression profile (Figure 1b). The transcription factors NKX2.5 [33] and GATA4 [34] are common to both populations, while the primary field additionally expresses TBX5 [35] and the secondary field expresses MEF2C [36] and ISL1 [37]. However, it should be noted that each of these factors is expressed in a broader domain and it appears to be the combination of factors that determine fate. For example, ISL1 is also expressed earlier in development in a proliferating cardiac progenitor population that gives rise to both first and second heart field lineages [38], while MEF2C is widely expressed in the embryo, its second heart field expression being driven by a specific cardiac enhancer [39].

It appears that one of the key events in cardiomyocyte specification is the repression of alternative muscle cell fates. The three contractile muscle types (cardiac, skeletal, and smooth) share many properties but commonly express different isoforms of related genes (e.g., myosins). Myocardin (MYOCD) is a transcriptional co-activator of the ubiquitously expressed transcription factor serum response factor (SRF) [40]. It is expressed in cardiac progenitors and in vascular smooth muscle. MYOCD is negatively regulated by the micro RNAs miR1 and miR133a, which are expressed in cardiac and skeletal muscle. High levels of MYOCD drive specification of smooth muscle in preference to cardiomyocytes [41].

Unlike cardiomyocytes, both cardiac fibroblasts and vascular smooth muscle may be derived from multiple lineages, which converge on a common cell fate (Figure 1a). Perhaps for this reason, both cell types are highly plastic and show an ability to transdifferentiate to a migratory state under certain conditions. Cardiac fibroblasts are a poorly defined population of cells of multiple embryonic origins [42]. About 80% of cardiac fibroblasts residing within the myocardium are derived from the outer layer of the heart, the epicardium, while the rest are largely derived from the endocardium [43,44], specifically from valve endothelium. The epicardium is derived from the intermediate mesoderm lineage and appears to share a common origin and gene expression profile to the kidney [45], for example in expression of the Wilm’s tumour transcription factor WT1. Multipotent progenitor cells known as epicardium-derived cells (EPDCs) migrate into the heart in mid gestation where they differentiate into multiple cell types including fibroblasts, endothelium, and smooth muscle [45]. In contrast, the endothelium is derived from the lateral plate mesoderm and shares a common origin with haematopoietic lineages, both being derived from a common precursor, the haemangioblast [46,47].

A minority of cardiac cells arise from an extra-mesodermal origin. The ectoderm lineage forms the neural crest, a population of multipotent progenitor cells that arises on the edge of the neural plate and migrate to various parts of the body. The cardiac neural crest migrates into the forming heart tube to populate the outflow tract and contributes to smooth muscle and vascular endothelium [48].

## 3. Cellular Events Resulting from Myocardial Infarction

### 3.1. Initial Inflammatory Response

Occlusion of a coronary artery leads to ischaemic myocardial cell death. Death occurs by both necrosis and apoptosis and is observed both within the infarct itself and within the surrounding region. Cell death peaks within the first 24 h after infarction, although low levels of cell death persist for several months as cardiac remodelling progresses [49].

An inflammatory response leads to activation of the innate immune system within minutes of an infarction. Necrotic cardiomyocytes themselves release a number of signals into the circulation collectively known as damage-associated molecular patterns (DAMPs) [18]. Inflammatory cytokines are also produced by a range of resident cells including macrophages, mast cells, fibroblasts and surviving cardiomyocytes. For example, cardiomyocytes produce interleukin-6 [50], while macrophages produce CXCL8 [51]. These signals serve to recruit circulating neutrophils and monocytes into the heart, where they differentiate into macrophages and phagocytose necrotic cells.

After several days, inflammation is reduced, neutrophil numbers drop, and the injured heart enters a reparative phase, mediated by macrophages [18]. Macrophages release VEGF to promote angiogenesis and TGFβ, which has many functions but crucially activates and recruits fibroblasts into the heart [52]. Thus, this is a temporary phase, but initiates the start of fibrosis, leading to permanent changes in the heart.

### 3.2. Fibrosis

Primitive vertebrates such as the zebrafish [53,54], together with neonatal mammals [55], are able to regenerate the heart following injury. In these cases, little or only transient scarring is observed prior to regeneration, and a dampened immune response is seen. In contrast, the adult mammalian heart is unable to regenerate and therefore a damage limitation strategy is employed, which involves the process of fibrosis. Fibrosis is the replacement of dead contractile tissue with live connective tissue, fibroblasts, in order to maintain structural integrity. This repair, which is initially beneficial, becomes pathogenic when it results in uncontrolled deposition of ECM protein and scar tissue formation [56].

Cardiac fibroblasts are much less sensitive to ischaemia than cardiomyocytes and generally survive an infarction. In addition to endogenous cardiac fibroblasts, fibroblasts of multiple origins are recruited into the heart upon injury. This includes lineages that do not contribute cardiac fibroblasts during embryogenesis, such as haematopoietic bone marrow-derived cells [57,58].

Fibroblasts have a remarkable ability to undergo transdifferentiation. TGFβ signalling serves not only to recruit fibroblasts into the heart, but also acts to phenotypically transform them to become myofibroblasts [59]. These are cells that combine features of a fibroblast and a smooth muscle cell; they are able to secrete large amounts of extracellular matrix proteins such as collagen, but also have contractile ability through the expression of α-smooth muscle actin (ACTA2) microfilaments [60,61]. Myofibroblasts are normally absent in healthy myocardium, but become highly abundant shortly after injury [60]. Although it was previously thought that these cells are derived from the migratory fibroblasts recruited into the heart upon injury, it is now thought that the majority of activated myofibroblasts are derived from transdifferentiated resident cardiac fibroblasts from the epicardial lineage [16]. Transdifferentiation of fibroblasts is generally regarded as a two-step process. Fibroblasts initially differentiate into a highly proliferative form known as an activated fibroblast [16]. These cells contribute to the innate immune response removing damaged tissue through both the recruitment of monocytes (by secretion of inflammatory cytokines and chemokines), and secretion of matrix metalloproteinases, which act to degrade the extracellular matrix, releasing damaged or dead cardiomyocytes for phagocytic clearance [62]. While it was previously believed that scar tissue collagen is deposited only by activated myofibroblasts, recent genomics analysis performed in the mouse suggests that macrophages also directly contribute collagen [63].

### 3.3. Progression to Heart Failure

Heart failure is a progressive loss of efficiency of the heart. Structural and functional remodelling of the heart as a response to stress and can be seen, for example, in response to exercise. However, pathological remodelling is a response to disease and results in deposition of extracellular matrix coupled with cardiomyocyte necrosis [64]. Although scar formation is initially beneficial to the heart, persistent myofibroblasts lead to an accumulation of fibrous tissue [15]. This, in turn, leads to progressive adverse myocardial remodelling [15]. Heart failure due to pathological fibrotic remodelling is a major cause of mortality in survivors of myocardial infarctions [65,66], and this is the reason why regeneration of the myocardium and reversal of fibrosis is so important.

## 4. Regenerative Medicine

Regenerative medicine holds much promise for repair of the injured heart. Reprogramming through forced expression of transcription factors has been shown to reverse epigenetic silencing of the genome leading to a state of induced pluripotency (Section 4.1). The same technology has been adapted to directly reprogramme somatic cells from one fate to another, bypassing the pluripotent state (Section 4.2). Such approaches raise the possibility that cardiac fibroblasts within post-infarct scar tissue could be converted into cardiomyocytes to reverse pathological remodelling. In this section, we will first describe the principles of induced pluripotency before going on to discuss direct reprogramming in the heart.

### 4.1. Induced Pluripotent Technology

Pluripotency in the embryo is determined and maintained, not by a single transcription factor, but by a network of transcription factors that includes at its core OCT4, SOX2, and NANOG [67]. The network appears to operate by synergistic mutual activation of members of the core network together with repression of genes promoting differentiation. The three core factors cooperatively bind to regulatory elements of target genes [67]. Multiple levels of pluripotency exist spanning the range from naïve to primed pluripotency [68], this latter state being that of the post-implantation epiblast.

As development proceeds, these cells lose potency to become firstly multipotent stem cells such as the cardiac progenitors described above and ultimately to become fully differentiated cells. Fate specification was for a long time considered to be an irreversible process, as described in Waddington’s famous analogy of a ball rolling downhill through an epigenetic landscape of bifurcating valleys [69]. This notion was overturned in 2006 when it was shown that a differentiated cell type (mouse fibroblasts) could be “reprogrammed” back to a pluripotent state [4]. Takahashi and Yamanaka screened a panel of 24 candidate genes (selected either for their expression in embryonic stem cells or oncogenic properties) for their ability to reprogramme. Retroviral expression of all 24 factors successfully induced pluripotency as demonstrated by upregulation of an Fbx15 reporter gene. By progressively eliminating factors, Takahashi and Yamanaka were able to identify a minimum network of just four factors required for reprogramming, which have become known as the Yamanaka or OKSM factors: the ESC transcription factors OCT3/4 and SOX2, and the oncogenes cMYC and KLF4. These same factors were subsequently shown to reprogramme human dermal fibroblasts to a pluripotent state [70].

Thus, as is the case for embryonic cardiogenic specification, there is no single factor determining pluripotency, but rather a network of synergistic interacting factors performs this function. The precise makeup of the network appears to be somewhat flexible, as demonstrated by the observation that the oncogenes MYC and KLF4 can be substituted for NANOG and LIN28A [71]. The requirement for LIN28A is particularly interesting because this gene is not a transcription factor, but rather has a post-transcriptional function. LIN28A is an RNA-binding protein that can regulate both the processing of micro RNA (mIR) from precursors and the translation of coding mRNAs [72].

A number of groups have improved upon the original reprogramming methods, particularly in order to improve safety and translational clinical potential by reducing the potential for tumour formation. Methods include the use of non-integrating episomal plasmids derived from the Epstein-Barr virus [73,74], the use of mRNA [75], or the use of small molecules [76].

### 4.2. Direct Reprogramming of Cardiac Fibroblasts

**Figure 2:**
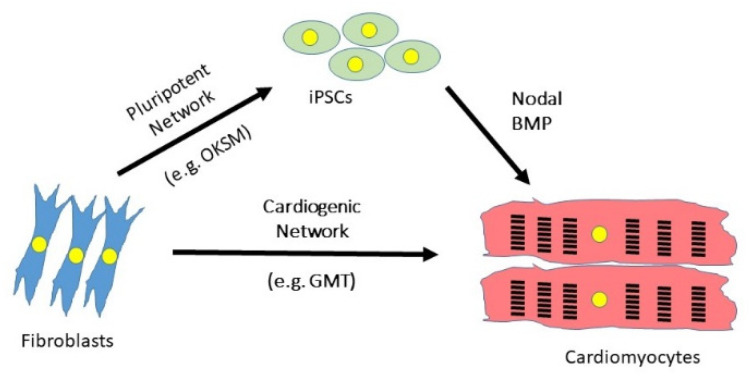
**Strategies to differentiate fibroblasts into cardiomyocytes.** Synergistic networks of transcription factors may be used either to reprogramme fibroblasts back to a pluripotent state from which cardiomyocytes can be differentiated by recapitulating embryonic development, or by direct reprogramming from one differentiated state to another. OKSM = OCT3/4, KLF4, SOX2, cMYC; GMT = GATA4, MEF2C, TBX5.

The work of Yamanaka’s group and others opened the door to personalised medicine. It was now possible, in theory at least, to take a skin biopsy from a patient and use this to generate an induced pluripotent stem cell (iPSC), which could then be differentiated in vitro into cardiovascular cell types using growth factors present in the embryo such as Nodal/Activin, FGF2 and BMP4 [77] (Figure 2). Such differentiated cells can be seeded onto a decellularised scaffold to generate a patch of tissue that may grafted during surgery. This has proven useful in the repair of some congenital heart diseases such as ventricular septal defect or valve replacement [78]. Many groups are also investigating the use of such patches to repair myocardial infarction. For example, Wang et al. seeded human iPSC-derived cardiomyocytes and fibroblasts onto a decellularised matrix and demonstrated that this patch reduced infarct size and improved cardiac function in a rat model of MI [79]. Su et al. used a microfluidics approach to engineer a network of blood vessels made from human umbilical cord endothelial cells, before seeding this with iPSC-derived cardiomyocytes [80]. An alternative approach, and the subject of this review, is that cardiac fibroblasts residing within the scar tissue itself might be reprogrammed in vivo to achieve regeneration of the injured myocardium.

To reverse the process of lineage specification and revert a cell to a state of pluripotency requires that heterochromatin be converted back to euchromatin through demethylation of DNA and acetylation of histone. Direct reprogramming or transdifferentiation from one differentiated cell type to another, bypassing the pluripotent stage, requires slightly different chromatin remodelling. Heterochromatic regions of the genome specifying the desired cell fate must be selectively opened up, while those specifying the old fate must be silenced. Such binary switches in cell fate are observed in the embryo, for example, the gene regulatory network comprising MYOCD, miR1, and miR133a, appears to control the selection of one of the muscle fates (cardiac, smooth or skeletal) through repression of alternative cell fates [41] (discussed above).

Fibroblasts are particularly attractive targets for direct reprogramming efforts because they are a naturally plastic cell type with the ability to transdifferentiate under certain conditions (Section 3.2). Cardiac fibroblasts, in addition, share considerable gene expression with cardiac progenitors, including many transcription factors of the cardiogenic network [17].

### 4.3. Identification of a Cardiogenic Network in Mouse Models

Ieda et al. [81] from Deepak Srivistava’a lab used a strategy similar to that previously used by Takahashi and Yamanaka by generating a transgenic reporter mouse that could be used to monitor fate specification. Ieda et al. chose to use MYH6 (α myosin heavy chain) as a marker of mature cardiomyocytes. The reader will recall that the regulation of the myosin heavy chain genes is complex. This family of genes demonstrates species and muscle type differences, in addition to being subject to developmental isoform switching [11,82]. In the human heart MYH6 is largely restricted to the atria while MYH7 is expressed in the ventricles, although there is some co-expression during embryonic development [83]. In mice, both MYH6 and MYH7 are co-expressed in the early heart tube, in later embryos the atrial/ventricular dichotomy seen in the human heart is transiently observed, before MYH6 becomes the dominant pan-cardiomyocyte marker in the postnatal heart [84]. Thus, it is not entirely correct that MYH6 expression is a reporter of the mature cardiomyocyte cell fate in mice as it is expressed as early as embryonic day 7.5 [84]. Nevertheless, this reporter has been subsequently used in many rodent studies (Table 1 and Table 2).

Ieda et al. [81] screened a pool of 14 transcription factors known to be expressed in cardiac progenitors for their ability to transdifferentiate fibroblasts. Ieda et al. gradually removed factors from this pool to identify the minimum combination sufficient for reprogramming. This work confirmed that MESP1 is insufficient for reprogramming, and indeed MESP1 is not present within the identified combination. Instead, they found that three factors GATA4, MEF2C, TBX5 (herein referred to as GMT) are: required. Thus, as in embryonic development, the cardiac lineage is not specified by a single factor, but by a synergistic network of cardiogenic factors. One interesting feature of this network is that it includes factors expressed largely in the first heart field (TBX5) and the second heart field (MEF2C) as well as one shared factor (GATA4). TBX5 has, however, been shown to be expressed in the posterior second heart field [85], where co-expression of all three factors occurs.

MEF2C and TBX5 have been shown to have properties of pioneer transcription factors, binding to regions of heterochromatin and facilitating chromatin remodelling to reactivate previously closed regions of the genome [86]. At the same time, enhancers active in fibroblasts are silenced [87]. MEF2C in particular appears to be a key element with MEF2 binding sites in the genome acting as sites of recruitment for multiple transcription factors [87]. In addition, the use of polycistronic expression constructs, which drive higher expression of MEF2C over GATA4 and TBX5, seem to be more efficient at reprogramming [88].

Another feature of the network is the importance of a combinatorial code for a specific cell fate [86,87]. Each transcription factor of the network is expressed in multiple cell types during development and therefore it is the specific combination of factors bound at a given locus that result in a specific outcome. Synergistic binding of transcription factors to target genes has been demonstrated, for example, in MEF2C and TBX5 to the target MYH6 [89].

As was found to be the case for the pluripotent network, the cardiogenic network exhibits flexibility, and a number of groups have subsequently demonstrated that a number of different combinations of factors achieve similar results. It would be impossible to discuss each of these in detail in a single review, but we have summarised the strategies used by these groups in Table 1. Table 2 summarises the results of these experiments. Most reprogramming combinations have at their core the GMT factors identified by Ieda. The Olson lab discovered that addition of a fourth transcription factor, HAND2, to the reprogramming cocktail improved efficiency [90] and the resulting GMHT cocktail has subsequently been used with various modifications in subsequent studies by the Olson [91,92,93], Song [94], and Gearhart [95,96] labs. Protze et al. tested 120 different three factor combinations and found that many different triplets elicited some degree of reprogramming [97]. Seven factors were most commonly seen in these networks: GATA4, MEF2C, TBX5, MESP1, NKX2.5, and MYOCD [97].

Repression of the fibroblast cell fate through inhibition of either TGFβ or RhoA/ROCK signalling has been found to enhance the effectiveness of the cardiogenic network [92].

**Table 1:** **A summary of strategies used to reprogramme cells into cardiomyocytes**. Refer to Table 2 for details of reprogramming efficiencies obtained with these strategies. I = inhibition, A = activation.

Laboratory	Species	Reprogramming Factors																									Reference
		Transcription Factors										miRNA				Pathway Targeting											
		GATA4	MEF2C	TBX5	MESP1	MYOCD	HAND2	NKX2-5	ZNF281	ESRRG	ZFPM2	1	133a	208	499	PI3/AKT	JAK/STAT	WNT	FGF	VEGF	TGFβ	RhoA-ROCK	Notch	cAMP/PKA	Epigenetic	BECN1 shRNA	
Srivistava	Mouse	X	X	X																							Ieda 2010 [81]
	Mouse	X	X	X																							Qian 2102 [98]
	Human	X	X	X	X	X				X	X																Fu 2013 [99]
	Mouse	X	X	X														I			I						Mohamed 2017 [100]
Olson	Mouse	X	X	X			X																				Song 2012 [90]
	Human	X		X		X	X					X	X														Nam 2013 [101]
	Mouse	X	X	X			X									A											Zhou 2015 [92]
	Mouse	X	X	X			X		X							A											Zhou 2017 [93]
	Mouse	X	X	X			X																I				Abad 2017 [91]
Ieda	Mouse	X	X	X																							Inagawa 2012 [102]
	Human	X	X	X	X	X																					Wada 2013 [103]
	Mouse	X	X	X									X														Muraoka 2014 [104]
	Human	X	X	X	X	X																					
	Mouse	X	X	X															A	A							Yamakawa 2015 [105]
Dzau	Mouse											X	X	X	X		I										Jayawardena 2012 [106]
Gearhart	Mouse	X	X	X			X	X																			Addis 2013 [95]
	Mouse	X	X	X			X	X													I						Ifkovits 2014 [96]
Song	Mouse	X	X	X			X					X	X								I	I					Zhao 2015 [94]
Ravens	Mouse		X	X		X																					Protze 2012 [97]
Qian	Mouse	X	X	X																							Wang 2015 [88]
	Mouse	X	X	X																						X	Wang 2020 [5]
	Human	X	X	X									X														Garbutt 2020 [107], Zhou 2019 [108]
Xie	Mouse																	A			I			A	A		Fu 2015 [109]
Kamp	Mouse	X		X	X			X																	A		Lalit 2016 [110]
Leong	Human	X	X	X		X		X				X	X														Christoforou 2017 [111]
Wu	Mouse	X	X	X																							Chen 2012 [112]

**Table 2:** Reprogramming efficiencies of methods used to reprogramme cells into cardiomyocytes. Refer to Table 1 for details of reprogramming strategy used. MEF = mouse embryonic fibroblast.

Laboratory	Species	In vitro/In Vivo	Source Cell	Developmental Stage	Reprogramming Efficiency	Comments	Reference
Srivistava	Mouse	In vitro	Cardiac Fibroblast	Postnatal	20% express MYH6 at 10 days	Although transdifferentiation is rapid, maturation (gain of TNNT2) takes several weeks	Ieda 2010 [81]
	Mouse	In vivo	Cardiac Fibroblast	Adult	10–15%	Cells are more mature than those reprogrammed in vitro	Qian 2012 [98]
	Human	In vitro	Cardiac Fibroblast	Foetal	20% express MYH6	Report that GMT alone cannot reprogramme human cells.	Fu 2013 [99]
			Dermal fibroblast	Neonatal			
			H9 ES-derived fibroblast	n/a			
	Mouse	In vitro	Cardiac Fibroblast	Neonatal	30% express MYH6 at 2 weeks	Almost doubles efficiency over GMT alone	Mohamed 2017 [100]
Olson	Mouse	In vitro	Cardiac fibroblast	Adult	6.8% express both MYH6 and TNNT2	Efficiency is 1.4% with GMT alone	Song 2012 [90]
			Tail tip Fibroblast	Adult	9.2% express both MYH6 and TNNT2		
		In vivo	Cardiac Fibroblast	Adult	At least 10,000 cells transdifferentiated	Improves heart function following infarction	
	Human	In vitro	Cardiac Fibroblast	Adult	13% express TNNT2	Sarcomere structures and calcium transients seen at 4–11 weeks	Nam 2013 [101]
			Dermal fibroblast	Adult	9.5% express TNNT2		
	Mouse	In vitro	MEF	Embryo	~25% express both MYH6 and TNNT2	Reprogramming more efficient in embryonic than adult cells.	Zhou 2015 [92]
			Tail tip fibroblast	Adult	~5% express both MYH6 and TNNT2		
			Cardiac Fibroblast	Adult	~6% express both MYH6 and TNNT2		
	Mouse	In vitro	Tail tip fibroblast	Adult	~28% express both MYH6 and TNNT2 after 7 days	Suppresses inflammatory signalling	Zhou 2017 [93]
	Mouse	In vitro	MEF	Embryo	Up to 70% express MYH6 and TNNT2 or ACTN2	Improves efficiency of generation of mature cardiomyocytes by GMHT by 5–6 fold	Abad 2017 [91]
Ieda	Mouse	In vivo	Cardiac Fibroblast	Adult	3% express MYH6 at 1 week	Reprogramming efficiency is lower than in other mouse in vivo studies	Inagawa 2012 [102]
	Human	In vitro	Cardiac Fibroblast	Adult	5% express TNNT2 and ACTN2 at 4 weeks	Report that GMT alone cannot reprogramme human cells.	Wada 2013 [103]
	Mouse	In vitro	MEF	Embryo	~35% express MYH6 at 1 week	Cardiomyocytes mature more quickly than GMT alone	Muraoka 2014 [104]
	Human	In vitro	Cardiac Fibroblast	Adult	~20% express TNNT2 at 1 week		
	Mouse	In vitro	MEF	Embryo	~70% beating cells at 4 weeks		Yamakawa 2015 [105]
Dzau	Mouse	In vitro	Fibroblast		1.5–7.7% express MYH6 with miR alone, increasing to 28% with JAK inhibitor	JAK inhibition dramatically improves reprogramming using miR	Jayawardena 2012 [106]
	Mouse	In vivo	Cardiac Fibroblast	Adult	Induced cardiomyocytes represent ~1% of total and express TNNT2		
Gearhart	Mouse	In vitro	MEF	Embryo	~1.5% show calcium oscillations at 2 weeks	Developed a quantifiable calcium reporter to assay efficiency HNGMT reported to be >50-fold more efficient than GMT alone	Addis 2013 [95]
	Mouse	In vitro	MEF	Embryo	~15% show calcium oscillations at 2 weeks	TGFβ inhibition improves efficiency 5 fold over HNGMT alone	Ifkovits 2014 [96]
Song	Mouse	In vitro	MEF	Embryo			Zhao 2015 [94]
			Cardiac Fibroblast	Adult	Up to 18% express TNNT2 at 4 weeks. 2.5% beating at 5 weeks		
			Tail tip fibroblast	Adult	Up to 20% express TNNT2 at 4 weeks. 4% beating at 5 weeks		
Ravens	Mouse	In vitro	MEF	Embryo	2.5% express MYH6	Found that a number of triplet combinations can be used.	Protze 2012 [97]
Qian	Mouse	In vitro	Cardiac Fibroblast	Adult	~10% express MYH6, ~5% express TNNT2	Demonstrated that expression of GMT as polycistronic MGT improves efficiency	Wang 2015 [88]
	Mouse	In vitro	MEF	Embryo	~10% express TNNT2 (GMT)	Use of Sendai virus improves efficiency over retrovirus	Miyamoto 2018 [113]
		In vitro	Tail tip fibroblast	Postnatal	~22% express TNNT2 (GMHT)		
		In vivo	Cardiac Fibroblast	Adult	~1.5% express TNNT2 (GMT)		
	Human	In vitro	Cardiac Fibroblast	Adult	~4% express TNNT2 (GMTMM) ~15% express TNNT2 (GMTMM+miR133)		
	Mouse	In vitro (In vivo)	Cardiac Fibroblast	Adult		Becn1 shRNA knockdown improves GMT efficiency	Wang 2020 [114]
	Human	In vitro	H9 ES-derived fibroblast	n/a	40–60% express TNNT2 at 2 weeks	Very efficient streamlined cocktail for human cells	Garbutt 2020 [107]
	Human	In vitro	Cardiac Fibroblast	Adult	~40% express TNNT2 at 2 weeks		Zhou 2019 [108]
Xie	Mouse	In vitro	MEF	Embryo	14.5% express ACTN2, 9% MYH6 on day 24		Fu 2015 [109]
		In vitro	Tail tip fibroblast	Adult			
Kamp	Mouse	In vitro	Cardiac Fibroblast	Adult	~7.25 colonies/50,000 cells	This method generates proliferating cardiac progenitor cells	Lalit 2016 [110]
		In vitro	Lung fibroblast	Adult			
		In vitro	Tail tip fibroblast	Adult			
Leong	Human	In vitro	Dermal fibroblast	Adult	Not stated		Christoforou 2017 [111]
Wu	Mouse	In vitro	Cardiac Fibroblast	2–3 weeks	No MYH6 expression at 3 weeks	Data suggest GMT reprogramming is inefficient	Chen 2012 [112]
		In vitro	Tail tip fibroblast	Adult	No MYH6 expression but 35% express TNNT2 at 3 weeks		

### 4.4. Reprogramming Human Cells

Human cells have proven to be more refractory to reprogramming than mouse cells, and not all findings in the rodent model have been found to be directly applicable to man. In particular, the GMT combination, which works well in mice, has been found to be insufficient to reprogramme human fibroblasts [99,103]. This impasse was overcome in 2013 when three different labs published three different methods for reprogramming human cells. The Srivistava lab used a reprogramming network of seven transcription factors (GATA4, MEF2C, TBX5, MESP1, MYOCD, ESRRG, and ZFPM2) [99], the Olson lab used a network of four transcription factors (GATA4, TBX5, MYOCD, and HAND2) together with two micro RNAs (miR-1 and miR-133a) [101], whilst Ieda’s lab used a five factor network (GATA4, MEF2C, TBX5, MESP1, and MYOCD) [103]. Direct comparisons between the methods are difficult because each group used a slightly different method to assay reprogramming efficiency (Table 2).

### 4.5. In Vivo Reprogramming of the Injured Heart

Whereas most studies to date have been performed in vitro, the attraction of this technology is that it raises the possibility to reprogramme the injured heart in vivo, thus repairing the myocardium at the same time as reducing pathological remodelling. Reprogramming in vivo presents a new set of challenges, such as that of delivering the reprogramming factors to the correct cells and a much more variable cellular environment in which to induce such changes.

Four ground-breaking studies were published by different labs in close succession in 2012 (Table 1 and Table 2).

Qian et al. [98], working in Srivastava’s lab, and Inagawa et al. [102], working on Ieda’s lab, independently demonstrated in vivo reprogramming of murine cardiac fibroblasts by retroviral delivery of GMT following myocardial infarction induced by coronary artery ligation. Qian et al. found that the retrovirus delivered to the myocardium bordering the infarct zone on the same day as infarction can transduce proliferating vimentin positive activated fibroblasts in the injured heart, but not non-proliferating cells such as resident cardiac fibroblasts in the normal heart. Whilst this could be seen as a limitation on the efficiency of reprogramming, it is advantageous to be able to target reprogramming specifically to the cells participating in remodelling, reducing off-target effects. Lineage tracing using periostin CRE revealed that ACTN1 positive induced cardiomyocytes were indeed derived from fibroblasts. Qian et al. found that although reprogramming efficiency (10–15%) was similar to that observed in vitro, the induced cardiomyocytes produced were more mature in vivo and they had sarcomeric structures resembling endogenous cardiomyocytes. Statistically significant improvements in cardiac function were seen by 8–12 weeks in reprogrammed hearts relative to controls including in the ejection fraction, stroke volume and cardiac output.

Inagawa et al. [102] also transduced GMT using a retroviral vector immediately following coronary artery ligation. An MYH6 reporter was expressed in 3% of viral infected cells after 2 weeks, a rate much lower than observed by Qian et al., and again only proliferating cells were targeted. Improvements in cardiac physiology were not investigated in this study.

Song et al. [90], working in Olson’s lab, used a similar strategy to test in vivo reprogramming by injecting GMHT retrovirus immediately following coronary artery ligation. Lineage tracing using a different CRE line, the fibroblast-specific calcium-binding protein S100A4, confirmed transdifferentiation of proliferating fibroblasts. Assessment of cardiac physiology demonstrated a progressive reduction in fractional shortening and ejection fraction beginning 24 h after injury in controls. This loss of efficiency was reduced in GMHT hearts in the weeks following injury. By 12 weeks post-injury, stroke volume of GMHT treated animals exceeded that of unoperated controls, while ejection fraction approached this level. Thus, these animals showed a greater improvement in function than GMT treated animals. Fibrosis was also reduced.

Jayawardena et al. [106], working in Dzau’s lab, used a lentivirus to deliver four micro RNAs to the injured heart and were also able to demonstrate transdifferentiation of S100A4 CRE cells. This method did not improve cardiac function immediately after ligation but was shown to significantly improve fractional shortening and velocity of circumferential fibre shortening by 2–3 months after injury [115].

The above studies utilise viral vectors that raise safety concerns regarding insertional mutagenesis, limiting their clinical applications. In 2015, Miyamoto et al. [113], working in Ieda’s lab, tested the use of a non-integrating Sendai virus vector. This virus replicates in the cytoplasm and does not integrate into the host genome, expression of reprogramming factors is therefore transient and not inherited by daughter cells upon cell division. Surprisingly, Miyamoto et al. found that this vector improved both efficiency of reprogramming and maturation of the resulting cells when reprogramming using GMT. In addition, immunostaining revealed specific targeting of cardiac fibroblasts and not cardiomyoctes. Functional studies demonstrated significant improvements in ejection fraction and functional shortening relative to controls at 4 weeks as well as a significant reduction in fibrotic area. An alternative approach was taken by Chang et al., working in Kim’s lab, who used gold nanoparticles coated with an arginine-rich peptide in order to deliver GMT to the mouse heart following coronary artery ligation [116]. This work demonstrated reduced fibrotic area and infarct thickness two weeks after injury.

Thus, in summary, results from a number of different groups have independently shown that cardiac function post infarction can be improved by reprogramming. Indeed, reprogramming in vivo tends to produce more mature cardiomyocytes than in vitro efforts, perhaps because the induced cardiomyocytes receive as-yet unidentified signals from their cellular environment within the tissue [117].

One caveat of these studies is that the mice used tend to be very young. Qian et al. used mice at 2 months old, while Song et al. used mice of 8–10 weeks. As mice reach sexual maturity at 6 weeks, these mice are therefore the equivalent of teenagers or young adults, whereas in man myocardial infarction is generally a disease affecting older people. As we know the murine heart maintains an intrinsic regenerative capacity in the neonatal period [55], and that embryonic cells are generally more easily reprogrammed in vitro [92] it is likely that reprogramming efficiency would be lower in an older heart.

## 5. Discussion

In this review, we have shown how a detailed understanding of cell fate determination during cardiovascular embryonic development has been utilised to inform strategies to reprogramme cardiac fibroblasts in the injured heart. As was found to be the case in the embryo, there is not a single transcription factor that can flip a switch between two differentiated cell states but rather acquisition of a cardiomyocyte fate appears to depend on a synergistic interaction between multiple factors. This network appears to be somewhat tolerant of substitutions, but there are species-specific differences.

It is perhaps noteworthy that almost a decade after in vivo reprogramming was first reported in a mouse model of myocardial infarction, there are as yet no published clinical successes and no current studies are listed in clinicaltrials.gov. This is indicative of the many challenges that remain to be overcome in the field before this potential can be realised in the clinic [117,118]. Two key issues remain unsolved. These are the low rate of observed reprogramming and safety concerns regarding the use of integrating viral vectors.

Determining the level of reprogramming required to obtain a clinically relevant outcome for patients is a key question. Most studies report a relatively low rate of reprogramming even in the young heart (Table 2), raising concerns that this may be insufficient to improve cardiac function in elderly patients. Nevertheless, evidence from mouse models has suggested that a relatively low level of reprogramming can result in a measurable improvement in cardiac function. For example, Qian et al. reported a significant improvement in ejection fraction and stroke volume with a reprogramming rate of 10–15% [98], while Song et al. found that the stroke volume of treated animals exceeded that of unoperated controls at 12 weeks [90]. We do not yet know whether the same effect would be seen in a patient and some have suggested a much higher level of reprogramming is needed, perhaps as high as 50% [119]. Only the recently published streamlined method developed in the Qian lab has approached this level of efficiency, achieving a rate of 40–60% [107,108], but this is an in vitro method that utilises a selectable marker for infected cells limiting its potential for translation. Perhaps a fruitful avenue for future basic research would be to study the relationship between reprogramming efficiency and functional recovery in the mouse MI model, or a more clinically relevant animal model such as the pig, in order to put a more precise figure on the required reprogramming threshold to inform future efforts. We also need more detailed data on the long-term impacts of reprogramming. How long do reprogrammed cells survive, are they epigenetically compromised in some way? Furthermore, all such studies to date have studied reprogramming in an acute model of myocardial infarction, studies on chronic heart failure models are lacking [120].

How can reprogramming efficiency be improved in vivo? In simple terms, this involves ensuring that the full complement of reprogramming factors is delivered to as many cells as possible and that these cells are receptive to those factors. Reprogramming appears to be effective only for dividing rather than quiescent cells [53,90,98]. To date, the virus has been delivered at the same time as the coronary artery is ligated in all in vivo mouse studies, but it may be that efficiency could be improved by treatment at a later stage when activated fibroblasts are at the peak of their proliferation, before their transdifferentiation to the more quiescent myofibroblasts (Section 3.2). It will also be important to target these cells before harmful collagen deposition occurs. The best timepoint would need to be optimised. The use of polycistronic vectors improves delivery efficiency and also allows manipulation of the relative levels of each factor, which also appears to be an important consideration [88].

Cardiac fibroblasts perform an important role in the heart and therefore we must ensure that only harmful scar tissue is reprogrammed. How can we target reprogramming to specific cell populations to prevent harmful off-target effects? Perhaps the answer to this may lie in engineering vectors to drive expression using enhancers/promoters active only in activated myofibroblasts, or by building in negative selection, which shuts down the expression of reprogramming factors if present in an unwanted cell type. Viruses could be targeted to specific cell types by engineering surface epitopes.

Safety is a concern with any viral vector due to the danger of oncogenic insertional mutagenesis and this has been an impediment in the clinical application of this technology. Qian’s highly efficient method [107,108] nevertheless depends on the use of integrating viruses, which may be unsafe for clinical use, and development of a non-integrating approach is needed. To this end, results from Ieda’s lab in mice using the non-integrating Sendai virus are promising, and fortuitously this method also appears to improve reprogramming efficiency [113].

An alternative approach to improve safety may be to eliminate the viral vector entirely and to find alternative methods. Small molecule drugs can modify epigenetic status [109]. Chang et al. have demonstrated the successful delivery of GMT using gold nanoparticles coated with an arginine-rich peptide in a mouse model of MI [116]. However, these are non-specific methods that do not specifically target activated myofibroblasts and may result in harmful alterations to other cells in the heart. In addition, small molecule epigenetic modifiers may modify off-target gene loci.

A modification of CRISPR gene editing may provide a method to specifically modify the epigenetic status of endogenous genes required for reprogramming. A modified enzymatically inactive version of CAS9, known as dead CAS9 (dCAS9) can be targeted using short guide RNAs to enhancers of transcription factors such as the GMHT network [114]. The guide RNA contains aptamers that recruit transcriptional activators to the locus, while CAS9 itself can be engineered to contain an activation domain [121]. This method has the advantage of activating endogenous genes rather than driving transgene expression and is also more easily adaptable to target different genes in the cardiogenic network. However, expression of dCAS9 in the original protocol required viral transduction and an alternative non-viral delivery method needs to be developed.

miRNA has been shown to be effective in reprogramming [106]. Although in vivo work to date has used a viral vector to drive miRNA expression, it is possible to deliver miRNA to cells without the use of a virus. For example, miRNAs are known to be transferred between cells by extracellular vesicles and these can be harnessed as novel therapeutics [122]. miRNA or synthetic mimics can be loaded into vesicles produced in vitro and these could in theory be targeted to specific populations of cells within the injured heart by engineering of transmembrane proteins [123]. Such an approach may also be useful for delivery of drugs or mRNA.

Despite these hurdles that remain to be fully overcome the field offers much hope that one day a patient arriving at an intensive cardiac unit following an acute myocardial infarction may receive a simple injection as part of their treatment and this might serve to regenerate the myocardium, preventing fibrosis and restoring cardiac function.

## Data Availability

Not applicable.
